# Dichloroacetate as a metabolic modulator of heart mitochondrial proteome under conditions of reduced oxygen utilization

**DOI:** 10.1038/s41598-022-20696-5

**Published:** 2022-09-29

**Authors:** Natalia Andelova, Iveta Waczulikova, Lukas Kunstek, Ivan Talian, Tanya Ravingerova, Magdalena Jasova, Simon Suty, Miroslav Ferko

**Affiliations:** 1 Centre of Experimental Medicine, Institute for Heart Research, Slovak Academy of Sciences, 84104 Bratislava, Slovakia; 2 Division of Biomedical Physics, Department of Nuclear Physics and Biophysics, Faculty of Mathematics, Physics and Informatics, Comenius University, 84248 Bratislava, Slovakia; 3 Department of Medical and Clinical Biophysics, Faculty of Medicine, P. J. Safarik University, 04011 Kosice, Slovakia

**Keywords:** Mitochondria, Physiology

## Abstract

Myocardial compensatory mechanisms stimulated by reduced oxygen utilization caused by streptozotocin-induced diabetes mellitus (DM) and treated with dichloroacetate (DCA) are presumably associated with the regulation of mitochondria. We aimed to promote the understanding of key signaling pathways and identify effectors involved in signal transduction. Proteomic analysis and fluorescence spectroscopy measurements revealed significantly decreased membrane potential and upregulated protein amine oxidase [flavin-containing] A (AOFA) in DM mitochondria, indicative of oxidative damage. DCA in diabetic animals (DM + DCA) downregulated AOFA, increased membrane potential, and stimulated thioredoxin-dependent peroxide reductase, a protein with antioxidant function. Furthermore, the DM condition was associated with mitochondrial resistance to calcium overload through mitochondrial permeability transition pores (mPTPs) regulation, despite an increased protein level of voltage-dependent anion-selective protein (VDAC1). In contrast, DM + DCA influenced ROS levels and downregulated VDAC1 and VDAC3 when compared to DM alone. The diabetic myocardium showed an identical pattern of mPTP protein interactions as in the control group, but the interactions were attenuated. Characterization of the combined effect of DM + DCA is a novel finding showing that DCA acted as an effector of VDAC protein interactions, calcium uptake regulation, and ROS production. Overall, DM and DCA did not exhibit an additive effect, but an individual cardioprotective pathway.

## Introduction

The state of reduced oxygen utilization, which develops in the myocardium in the conditions of acute experimental streptozotocin-induced diabetes mellitus (DM), can be associated with the triggering of adaptive processes leading to paradoxically lower susceptibility to ischemia in experimental studies^1–4^. Activation of endogenous protective pathways lead to a positive remodeling of the mitochondrial membrane, acting against the increased energy demand associated with DM itself^5–8^. It has been proposed that DM shares some molecular pathways with ischemic preconditioning (PC) and with other forms of endogenous protection against ischemia/reperfusion (I/R) injury^1,2,9^. The lack of oxygen in DM is not caused by its absence but rather by a disruption of the respiratory chain of cardiac mitochondria, which impairs its ability to utilize it^9,10^. From a pathophysiological point of view, this state may be termed pseudohypoxia, a situation that resembles mild hypoxia, but with a considerable decrease in tissue pO_2_ and the accompanying switch over to lactate production being absent^6,11^. Hyperglycemia is often associated with pseudohypoxia, which is characterized by an increase in the ratio of nicotinamide adenine dinucleotide (NADH/NAD^+^)^12,13^. Under hypoxic conditions, the balance is disturbed via impaired oxidation of NADH. Some authors do not support the concept of pseudohypoxia because they did not observe changes in the amounts of NADH and NAD^+^^13^. Our results from previously published work demonstrated mitochondrial respiratory chain damage of the diabetic myocardium but without disconnection oxidation from oxidative phosphorylation and slowed-down electron flow associated with a reduction in the rate of oxidative phosphorylation during oxidation of succinate as well as glutamate as substrates ^6^. DM-induced impairment of electron transport in the respiratory chain is likely to be more pronounced in substrates whose oxidation occurs through a change in the NADH/NAD^+^ ratio. The metabolic alterations that are developing in the heart of experimental DM models affect mitochondrial energy transfer mechanisms leading to adaptation to increased energy requirements due to increased Ca^2+^ transitions, resulting in a reduced susceptibility of the diabetic heart to injury.

Because mitochondria play a vital role in oxygen metabolism^14^, it is necessary to monitor their performance with respect to diseases that are characterized by increased energy requirements^15^, such as diabetes mellitus^16,17^. Mitochondria appear to be crucial to maintain a sufficient supply of energy to the heart during pathological conditions^18,19^.

In connection with the compromised energy supply in hypoxic conditions of the acute phase of diabetic myocardial disease^20^, endogenous protective mechanisms associated with functional mitochondrial remodeling are activated at the level of chemical and physical properties of mitochondrial membranes^6,11^.

These attributes make this model a suitable candidate for studying cardiac mitochondria in conditions of reduced oxygen utilization. Understanding the signaling pathway mechanisms and the initiation of cardioprotective processes at the level of cardiac mitochondria opens new possibilities in connection with their therapeutic potential.

The effect of PC on the myocardium is associated with the initiation of a number of protective signaling pathways that control the activation of mitochondrial permeability transition pores (mPTPs)^9,21–23^. mPTPs do not constitute a classic membrane channel but rather a multiprotein complex whose identity has not yet been fully understood. mPTPs form and open upon Ca^2+^ increase in the mitochondrial matrix, elevation in reactive oxygen species (ROS), inorganic phosphate, and upon intracellular acidification. The processes of inhibiting mPTP opening, which might help secure the process of oxidative phosphorylation and thus the maintenance of adequate adenosine triphosphate (ATP) production, represent an essential and beneficial cardioprotective strategy^24,25^. The contribution to the regulation of the mPTP complex is important in terms of seeking promising potential targets^24,26,27^.

mPTPs appear to play a role in the mechanism of normal Ca^2+^ release required for proper metabolic regulation. It is hypothesized that transient opening of mPTPs may help regulate Ca^2+^ in the cytosol when Ca^2+^ overload occurs^28,29^. Transient opening of mPTP is also associated with a temporary increase in ROS, which serve as a signaling platform^29,30^, and with depolarization of the membrane potential in a small proportion of mitochondria, while a substantial proportion of mitochondria remain polarized and functional^31^. In contrast, unregulated mPTP opening causes oxidative damage, loss of inner membrane potential and disconnection of the respiratory chain^32^. Subsequently, these processes result in the arrest of mitochondrial ATP production, release of cytochrome c from mitochondria^30^, rupture of mitochondria, and cell death^33^.

In connection with the study of a pseudohypoxic experimental model, we used dichloroacetate (DCA), a substance acting as a pyruvate dehydrogenase kinase (PDHK) inhibitor, which regulates substrate metabolism in the heart. DCA treatment has been shown to reduce the infarction size caused by I/R^34,35^, to improve contractile dysfunction of cardiomyocytes, and to help control intracellular Ca^2+^ signaling under hypoxia/reoxygenation conditions. Another beneficial effect of DCA under these conditions was its effect on the reduction in ROS generation^36,37^.

However, the effect of DCA on mPTP regulation in relation to changes in mitochondrial membrane potential and ROS signaling has not yet been described. The aim of this study was therefore to monitor the effect of DCA on the mentioned parameters in connection with pseudohypoxic conditions induced by DM. Additionally, we aimed to verify how the endogenous protective mechanisms induced by DM might interact with the cardioprotective effects of DCA.

In addition to the identification of biophysical changes of mitochondrial membranes at the supramolecular level, we also studied changes in mitochondrial proteomics with the aim of identifying and characterizing molecular mechanisms that provided valuable information about pathological, adaptive and functional processes in the myocardium. The study of mPTPs at the proteomic level and the understanding of the importance of protein–protein interactions between the individual structural and regulatory components of mPTPs allowed us to gain a more detailed overview of the potential initiation and signaling pathways involved in compensatory mechanisms triggered by DM.

## Results

### Biochemical characterization of the experimental models

The effects of the experimental model of streptozotocin-induced diabetes (DM) and dichloroacetate (DCA) on the metabolic parameters are shown in Table 1. The average starting weight of the rats was 302 ± 14 g. The weight of the diabetic animals was significantly lower by approximately 14% on average in comparison with healthy control animals (Control) before the measurements. The animal's disease status was confirmed on Day 8 by statistically significant increases in plasma glucose, triacylglycerols, and blood cholesterol, as well as a significant reduction in blood insulin levels compared to the similarly aged Control. Body weights and metabolic parameters were not significantly different between groups unaffected and affected by DCA.

**Table 1:** Metabolic state and body weight of the experimental rats.

Metabolic parameters and body weight	Control	DCA	DM	DM + DCA
Glucose (mmol.L^−1^)	6.78 ± 0.36	5.80 ± 0.21	26.3 ± 2.18**	25.2 ± 2.06**
Triacylglycerol (g.L^−1^)	1.40 ± 0.22	1.33 ± 0.18	4.92 ± 0.43**	4.85 ± 0.38**
Cholesterol (mmol.L^−1^)	1.85 ± 0.26	1.79 ± 0.19	2.54 ± 0.30*	2.52 ± 0.24*
Insulin (ng.mL^−1^)	1.11 ± 0.18	1.13 ± 0.20	0.42 ± 0.10**	0.46 ± 0.14**
Body weight (g)	360 ± 12	368 ± 14	319 ± 19*	307 ± 18**

All values are presented as the mean ± standard error of the mean (SEM). Control—untreated control group; DCA—control group treated with dichloroacetate; DM—diabetic group; DM + DCA—diabetic group treated with dichloroacetate *p < 0.05, **p < 0.01 compared to Control.

### Fluorescence analyses—characterization of mitochondrial membrane properties

#### Mitochondrial calcium retention capacity

**Figure 1:**
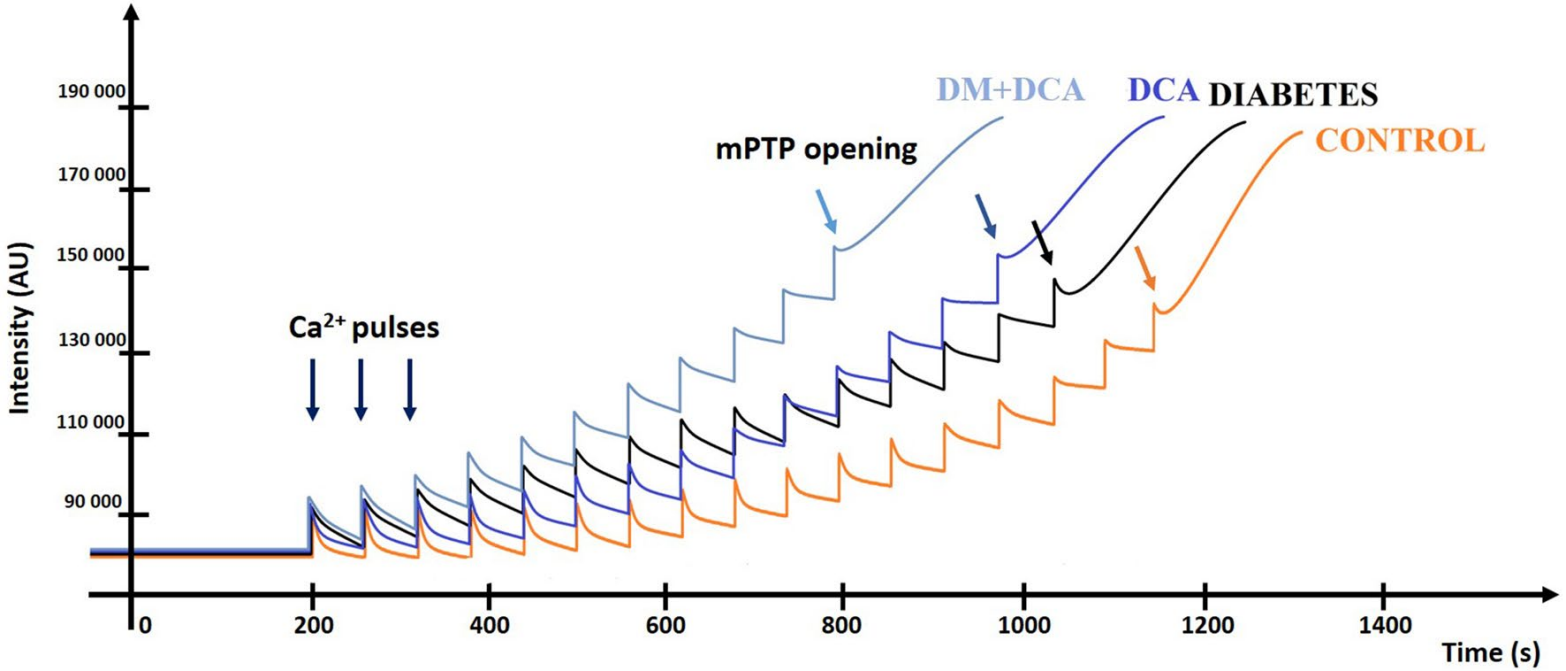
Mitochondrial CRC performed on isolated cardiac mitochondria using the fluorescent probe Calcium Green™-5 N—representative graphs of all experimental groups. CaCl_2_ pulses (0.125 mM/injection) were added at 60 s intervals after 200 s stabilization. Ca^2+^-induced mPTP opening was observed after the fluorescence intensity increased.

**Figure 2:**
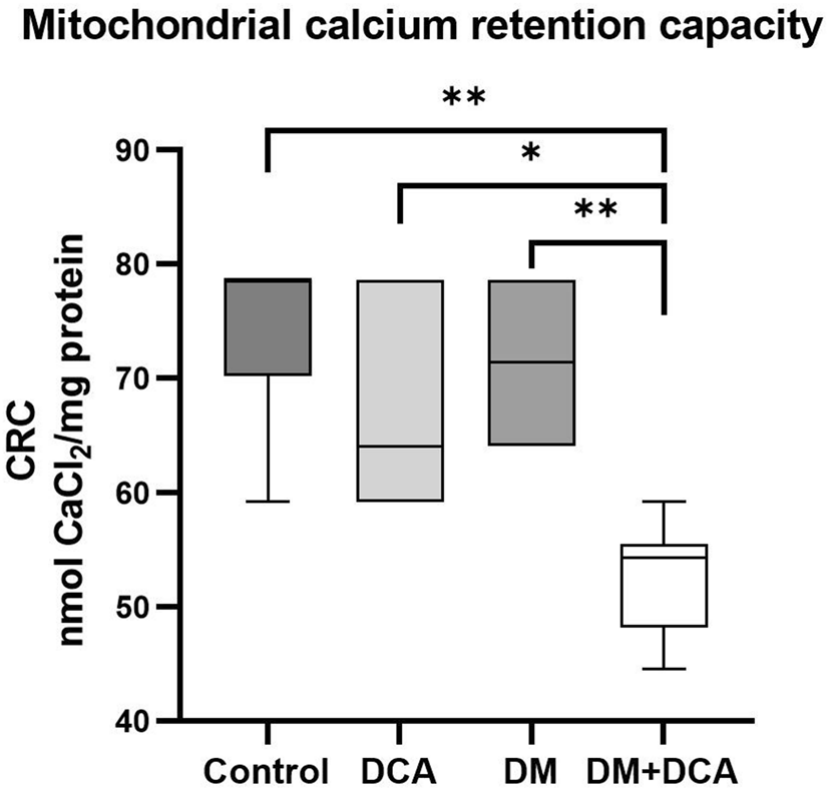
Changes in the CRC of isolated cardiac mitochondria induced by DCA treatment and by experimental DM conditions*.* CRC was maintained in the DM group versus the Control group. DCA application in the DM group significantly reduced CRC. Control—untreated control group; DCA—control group treated with dichloroacetate; DM—diabetic group; DM + DCA—diabetic group treated with dichloroacetate. Box plot graph displays minimum and maximum values and median shown by a horizontal line. n = 6 per group; *− *p* < 0.05; **− *p* < 0.01. Control versus DM + DCA (*p* = 0.0012), DCA versus DM + DCA (*p* = 0.0268), DM versus D + DCA (*p* = 0.0047).

Mitochondrial calcium retention capacity (CRC) characterizes the sensitivity of isolated mitochondria to Ca^2+^ and is directly related to delaying mPTP opening. More specifically, CRC is defined as the amount of total Ca^2+^ that has to be accumulated inside the mitochondria to induce a permeable transition. This amount is expressed in nanomoles of CaCl_2_ per milligram of mitochondrial proteins. Representative traces of CRC measurements for all experimental groups are presented in Fig. 1. In the Control group, 74.61 ± 3.179 nmol CaCl_2_/mg protein was sufficient to induce mPTP opening (Fig. 2). Compared to the Control group, we observed a significant decrease of 30% in mitochondrial Ca^2+^ uptake only in the diabetic group affected by DCA (DM + DCA). The amount of added CaCl_2_ leading to mPTP opening was significantly reduced by 26% after DCA administration in the diabetic group (DM + DCA) (Fig. 2).

#### Mitochondrial membrane potential

**Figure 3:**
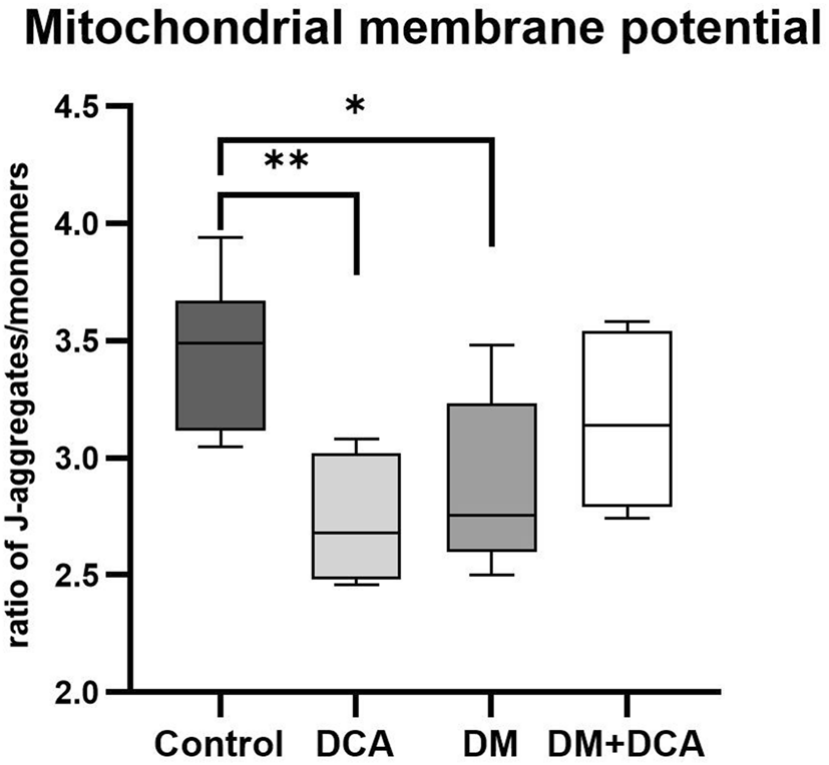
Effect of DCA treatment and experimental DM conditions on the mitochondrial membrane potential in the rat heart. Mitochondrial membrane potential was determined using the fluorescent probe JC-1 and expressed as the ratio of J-aggregates and monomers. The ratio of J-aggregates and monomers in the DCA group and DM group was significantly reduced compared with that in the Control group. Control—untreated control group; DCA—control group treated with dichloroacetate; DM—diabetic group; DM + DCA—diabetic group treated with dichloroacetate. Box plot graph displays minimum and maximum values and median shown by a horizontal line. n = 7 per DCA group, n = 8 per Control, DM, and DM + DCA group; *− *p* < 0.05, **− *p* < 0.01. Control versus DCA (*p* = 0.0030), Control versus DM (*p* = 0.0200).

Maintenance of mitochondrial membrane potential is required for normal energy production by functional mitochondria. The JC-1 fluorescent probe, which accumulates in the mitochondria in the form of monomers and J-aggregates, was used to determine the mitochondrial membrane potential. The ratio of the fluorescence intensity of J-aggregates and monomers represents a measure of the transmembrane potential. The value of the J-aggregate/monomer ratio in the Control group was 3.44 ± 0.11. We observed a significant decrease in the mitochondrial membrane potential by 20.35% in the Control group after DCA administration (DCA) and by 16.30% in the DM group compared to the Control group (Fig. 3). The effect of DCA in the DM group (DM + DCA) increased the mitochondrial membrane potential nearly at the level of the Control group. 2,4-dinitrophenol administration as a positive control confirmed a significant decrease in the ratio of J-aggregates and monomers in all experimental groups (data not shown).

**Figure 4:**
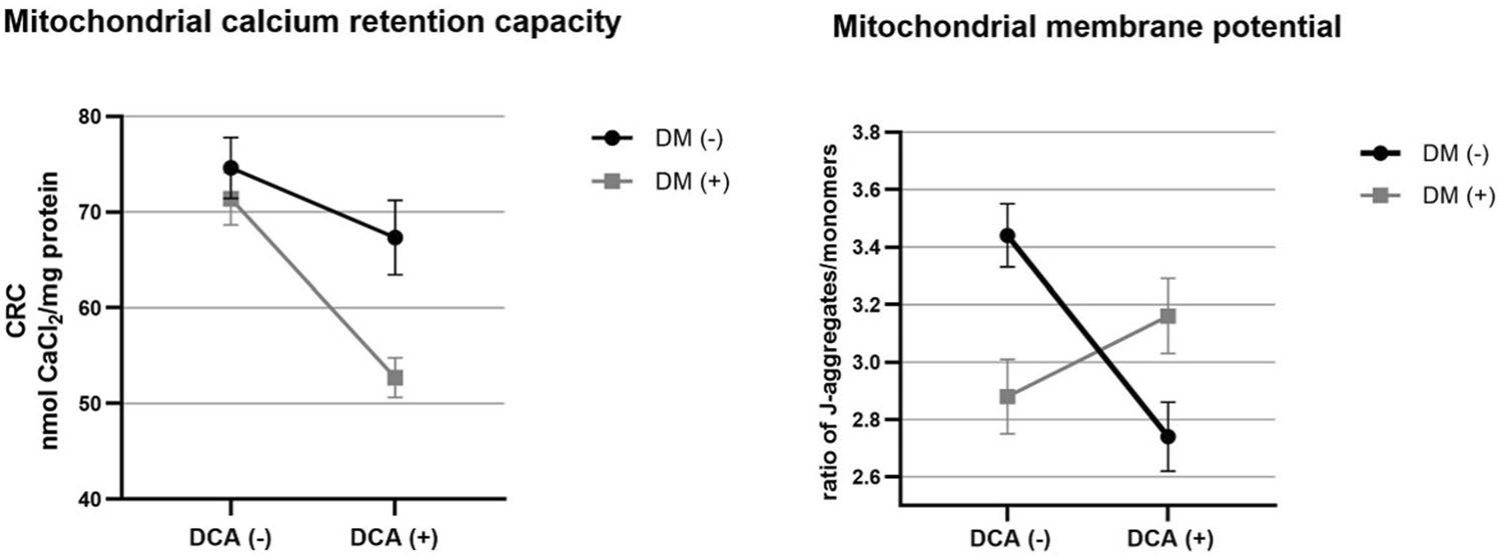
Graphs of the statistical interaction effect between the DCA treatment as the first main factor and experimentally induced diabetic (DM) condition as the second main factor. Both factors had two levels: exposed to DCA (DCA ( +)) or not exposed to DCA (DCA ( −)), and presence of diabetes (coded DM ( +)) or absence of diabetes (DM ( −)). Combinations of these 2 × 2 levels define all four treatment groups (the design is termed a full factorial design in two levels): “DCA ( −) and DM ( −)“ stands for healthy control group (Control); “DCA ( −) and DM ( +)“ stands for diabetic group (DM); “DCA ( +) and DM ( −)“ stands for healthy dichloroacetate group (DCA); and “DCA ( +) and DM ( +)“ stands for diabetic-dichloroacetate group (DM + DCA).

To determine whether group factor—experimental DM and treatment—DCA have an impact on the mitochondrial CRC and mitochondrial membrane potential, we chose a factorial experiment. We also tested the statistical interaction between both factors—experimental DM and the drug DCA—to determine what proportion of total variance in mitochondrial CRC and mitochondrial membrane potential data could be attributed to DM and to the effect of DCA. Two-way ANOVA yielded a significant effect of both main factors, experimental DM and DCA, on mitochondrial CRC but not on mitochondrial membrane potential. The latter finding is a direct consequence of the observed interaction between both main factors (*p* = 0.0002). For CRC, the statistical interaction test did not pass the significance threshold (*p* = 0.0761) (Fig. 4). The nonparallel course of the lines indicates, however, that the factors might not be additive in the joint effect, but the effect size of one factor represented by DCA depended on the level of the second factor represented by experimental DM. Thus, the main effects of factors DM and DCA should not be considered in isolation.

### Liquid chromatography and mass spectrometry-based proteomics

In the proteomic analyses, we focused on the mitochondrial proteins that are considered structural and/or regulatory components of the mPTP complex and 3 proteins related to oxidative damage or antioxidant potential, and they met strict quantification criteria.

#### Between-group differences in the protein abundance of the mPTP complex

**Figure 5:**
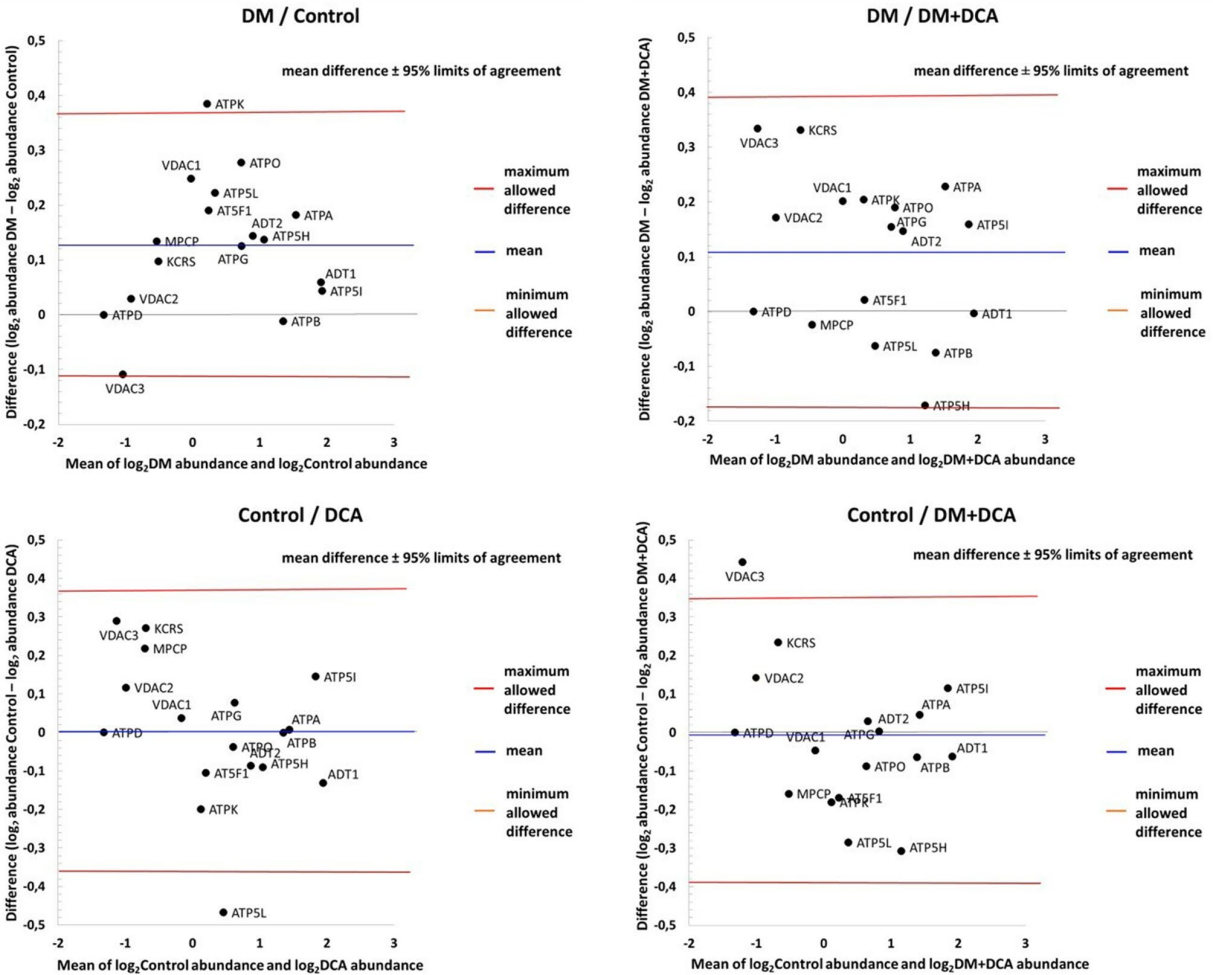
Scatter diagrams of the difference between the abundances of identified proteins involved in the structure and regulation of mPTPs in the two different experimental conditions plotted against the average of the two measurements, both on a log scale. We compared the differences between Groups DM versus Control (graph top left), DM versus DM + DCA (graph top right), Control versus DCA (graph bottom left) and Control versus DM + DCA (graph bottom right). Horizontal lines are drawn at the mean difference and at the limits of agreement (DM vs. Control: 95% LoA − 0.1099 to 0.3634; DM vs. DM + DCA: 95% LoA − 0.1795 to 0.3919; Control vs. DCA: 95% LoA − 0.3607 to 0.3662; Control vs. DM + DCA: 95% LoA − 0.3872 to 0.3461). The line of the mean clearly shows an absolute systematic difference (0.1268 for DM versus Control; 0.1062 for DM versus DM + DCA; 0.0028 for Control versus DCA and − 0.0205 for Control versus DM + DCA) for the investigated mPTP protein components (n = 17) in the two conditions (paired *t test p value* = 0.0005 for DM vs. Control; *p value* = 0.0084 for DM vs. DM + DCA; *p value* = 0.9517 for Control vs. DCA and *p value* = 0.6569 for Control vs. DM + DCA). The zero value denoted by the black dashed line corresponds to the line of equivalence. ADT1—ADP/ATP translocase 1; ADT2—ADP/ATP translocase 2; AT5F1—ATP synthase F(0) complex subunit B1; ATP5H—ATP synthase subunit d; ATP5I—ATP synthase subunit e; ATP5L—ATP synthase subunit g; ATPA—ATP synthase subunit alpha; ATPB—ATP synthase subunit beta; ATPD—ATP synthase subunit delta; ATPG—ATP synthase subunit gamma; ATPK—ATP synthase subunit f; ATPO—ATP synthase subunit O; KCRS—creatine kinase S-type; MPCP—phosphate carrier protein; VDAC1—voltage-dependent anion-selective channel protein 1; VDAC2—voltage-dependent anion-selective channel protein 2; VDAC3—voltage-dependent anion-selective channel protein 3.

First, we estimated the abundance of all identified mPTP proteins. The differences between the abundances of the mPTP proteins in the individual experimental conditions can be seen in Bland–Altman plots (Fig. 5). The subgroups of low-abundance proteins are centered on the left side, and those of high-abundance proteins are located on the right side. The mPTP proteins with the highest average abundance in all experimental groups were ADP/ATP translocase 1 (ADT1), ATP synthase subunit e (ATP5I), ATP synthase subunit alpha (ATPA), ATP synthase subunit beta (ATPB) and ATP synthase subunit d (ATP5H). The limits of agreement were defined as the mean difference ± 1.96 SD of difference. Based on the upper Bland–Altman plots (Fig. 5), the abundance of mPTP proteins as a whole was significantly different in the DM group compared to the healthy Control group (*p* = 0.0005) (graph top left) and DM + DCA group (*p* = 0.0084) (graph top right). The blue line in the upper graphs of Fig. 5 indicates significant mean upregulation—the whole region was above the zero value of no change. ATPA was the most abundant protein, which also achieved statistical significance in favor of the DM group (c.f., Table 2). In the case of the bottom graphs of Fig. 5, two experimental conditions were considered to be in agreement. The abundance of mPTP proteins as a whole was not significantly different in the Control group compared to the DCA group (*p* = 0.9517) (graph bottom left) and DM + DCA group (*p* = 0.6569) (graph bottom right).

**Table 2:** Analysis of the treatment effects on the logarithmic scale.

Protein	AVE LOG FC DM/Control	ANTILOG AVE FC DM/Control	*p* value	2-way RM ANOVA			AVE LOG FC DM/ DM + DCA	ANTILOG AVE FC DM/ DM + DCA	*p* value	2-way RM ANOVA		
				Treatment*(diabetes)	Run*	Interaction*				Treatment*(DCA)	Run*	Interaction*
**Identified proteins involved in the structure and regulation of mPTPs**												
ADT1	0.0571	1.0404	0.4834	0.4132	0.8809	0.6157	− 0.0022	0.9985	0.9641	0.9653	0.4191	0.9334
ADT2	0.1459	1.1064	0.1353	0.0773	0.7214	0.6299	0.1479	1.1080	0.1237	0.0758	0.9761	0.8784
AT5F1	0.1996	1.1484	0.2207	0.1870	0.8378	0.2739	0.0196	1.0137	0.9600	0.9542	0.3937	0.4676
ATP5H	0.1364	1.0992	0.2438	0.1741	0.4837	0.7052	− 0.1723	0.8874	0.1931	0.1275	0.8561	0.8974
ATP5I	0.0400	1.0281	0.7869	0.8261	0.1402	0.4508	0.1579	1.1157	0.2207	0.2516	0.7896	0.1343
ATP5L	0.2165	1.1619	0.2060	0.3588	0.1777	0.5036	− 0.0626	0.9576	0.4468	0.5173	0.2754	0.2720
ATPA	0.1824	1.1348	0.0231	0.0200	0.2807	0.9945	0.2385	1.1798	0.0131	0.0055	0.8264	0.2040
ATPB	− 0.0122	0.9916	0.8403	0.8057	0.2955	0.4380	− 0.0750	0.9493	0.2217	0.2836	0.1508	0.9389
ATPD	0.0000	1.0000	1.0000				0.0000	1.0000	1.0000			
ATPG	0.1252	1.0907	0.2374	0.2396	0.9023	0.8206	0.1592	1.1167	0.1238	0.1597	0.9659	0.7465
ATPK	0.3882	1.3088	0.0339	0.0236	0.4148	0.2834	0.2173	1.1626	0.3029	0.2359	0.4021	0.4927
ATPO	0.2763	1.2111	0.0312	0.0119	0.2251	0.4210	0.1891	1.1400	0.0969	0.0884	0.0840	0.9947
KCRS	0.0968	1.0694	0.4411	0.3763	0.8954	0.6606	0.3311	1.2580	0.0286	0.0130	0.5674	0.3798
MPCP	0.1335	1.0970	0.3109	0.2290	0.9365	0.2746	− 0.0166	0.9885	0.7348	0.7185	0.6215	0.0870
VDAC1	0.2562	1.1943	0.0329	0.0172	0.9359	0.3094	0.2034	1.1514	0.0225	0.0400	0.1454	0.6139
VDAC2	0.0307	1.0215	0.5172	0.6774	0.5226	0.9976	0.1749	1.1289	0.1884	0.1453	0.8898	0.7527
VDAC3	− 0.1114	0.9257	0.5286	0.4746	0.6969	0.2010	0.3476	1.2725	0.0237	0.0296	0.6061	0.3721
**Proteins related to oxidative damage / antioxidant potential**												
AOFA	1.0422	2.0594	< 0.0001	< 0.0001	0.2800	0.6953	0.4493	1.3654	0.0018	0.0057	0.9164	0.3684
PRDX3	− 0.0202	0.9861	0.8124	0.8505	0.6295	0.7143	− 0.3877	0.7643	0.0051	0.0009	0.6960	0.5957
PRDX5	− 0.0610	0.9586	0.6122	0.5703	0.6134	0.5220	0.1583	1.1160	0.4753	0.4499	0.6123	0.6325

Protein	AVE LOG FC Control/DCA	ANTILOG AVE FC Control/DCA	*p* value	2-way RM ANOVA			AVE LOG FC Control/ DM + DCA	ANTILOG AVE FC Control/ DM + DCA	*p* value	2-way RM ANOVA		
				Treatment*(DCA)	Run*	Interaction*				Treatment*(DM + DCA)	Run*	Interaction*
**Identified proteins involved in the structure and regulation of mPTPs**												
ADT1	− 0.1289	0.9145	0.1543	0.1649	0.6419	0.8683	− 0.0593	0.9597	0.2917	0.3962	0.8240	0.5683
ADT2	− 0.0821	0.9447	0.4569	0.3953	0.9546	0.5206	0.0020	1.0014	0.9595	0.9613	0.6542	0.7756
AT5F1	− 0.1193	0.9206	0.2490	0.3552	0.1928	0.7669	− 0.1800	0.8827	0.0571	0.0776	0.6004	0.5074
ATP5H	− 0.0905	0.9392	0.2420	0.3562	0.7173	0.1526	− 0.3088	0.8073	0.0064	0.0096	0.5978	0.6334
ATP5I	0.1392	1.1013	0.2832	0.2174	0.6767	0.8986	0.1179	1.0851	0.2934	0.2753	0.7076	0.3013
ATP5L	− 0.4695	0.7222	0.0352	0.0229	0.6399	0.7724	− 0.2791	0.8241	0.0609	0.0539	0.6445	0.6373
ATPA	0.0055	1.0038	0.9605	0.9753	0.5197	0.7277	0.0561	1.0397	0.5275	0.6448	0.8700	0.3218
ATPB	0.0003	1.0002	0.9923	0.9916	0.6250	0.8522	− 0.0628	0.9574	0.3331	0.3623	0.3974	0.5449
ATPD	0.0000	1.0000	1.0000				0.0000	1.0000	1.0000			
ATPG	0.0810	1.0578	0.6574	0.6936	0.5205	0.5839	0.0340	1.0238	0.7786	0.8830	0.8787	0.9464
ATPK	− 0.2049	0.8676	0.2066	0.2421	0.8209	0.9730	− 0.1709	0.8883	0.4839	0.4572	0.9764	0.8514
ATPO	− 0.0378	0.9741	0.5120	0.6362	0.3766	0.6651	− 0.0872	0.9413	0.5505	0.5430	0.3056	0.4994
KCRS	0.2721	1.2076	0.0888	0.0643	0.7394	0.3619	0.2343	1.1764	0.1558	0.1332	0.3824	0.7034
MPCP	0.2172	1.1625	0.0853	0.0857	0.2322	0.8736	− 0.1501	0.9012	0.0904	0.0877	0.0893	0.7103
VDAC1	0.0311	1.0218	0.6414	0.7358	0.4527	0.7970	− 0.0528	0.9641	0.7263	0.6478	0.7021	0.1620
VDAC2	0.1162	1.0839	0.4256	0.3582	0.8843	0.6476	0.1442	1.1051	0.3565	0.2777	0.9006	0.7744
VDAC3	0.3198	1.2481	0.1294	0.2288	0.4864	0.8664	0.4591	1.3747	0.0237	0.0125	0.5940	0.8591
**Proteins related to oxidative damage/antioxidant potential**												
AOFA	− 0.0387	0.9736	0.9632	0.9562	0.7638	0.3912	− 0.5930	0.6630	0.0055	0.0017	0.7748	0.2765
PRDX3	− 0.1513	0.9005	0.1055	0.1438	0.8213	0.4641	− 0.3676	0.7751	0.0026	0.0013	0.9592	0.3221
PRDX5	0.3322	1.2589	0.0458	0.0626	0.4303	0.3470	0.2193	1.1642	0.2173	0.1740	0.9607	0.9352

*p value from testing the hypothesis of no difference in log emPAI values for the indicated sources of variation compared to random variability. *FC* fold change; *AVE* average; P: probability; *RM* repeated measures; *AOFA *amine oxidase [flavin-containing] A; *PRDX3* thioredoxin-dependent peroxide reductase; *PRDX5* peroxiredoxin 5. Names and abbreviations of the proteins involved in the structure and regulation of mPTPs are in the legend of Fig. 5.

#### Individual protein expression (upregulation and downregulation of relevant proteins)

We further proceeded to identify changes in expression by individually analyzing mPTP proteins and proteins related to oxidative damage and antioxidant potential. The protein expression levels were presented as ratios of protein abundances of two groups and referred to as a fold change (FC). These results are shown in Table 2. Using two-way repeated measures (RM) ANOVA, we confirmed the significance of the treatment effect.

Individually analyzed proteins involved in the structure and regulation of the mPTP complex within the DM group, except for the three ATP synthase subunits ATPA, ATP synthase subunit f (ATPK) and ATP synthase subunit O (ATPO), and voltage-dependent anion-selective channel protein 1 (VDAC1), did not show significantly altered protein levels. The remaining quantified proteins were maintained without significant changes at the level of healthy mitochondria. The level of ATP synthase subunit delta (ATPD) protein was preserved without change in all four experimental groups. The most significant change in protein levels due to pseudohypoxic conditions induced by experimental DM was observed for amine oxidase [flavin-containing] A (AOFA) protein, which is associated with oxidative damage. Administration of DCA in the DM group slightly decreased the expression of mPTP proteins. At the level of ATPA, creatine kinase S-type (KCRS), VDAC1 and VDAC3 proteins were significantly downregulated in the DM + DCA group compared to the DM group. The drug DCA in the DM group reduced the protein level of AOFA, but the expression was still significantly increased in the DM group compared to the DM + DCA group. The effect of DCA in the DM group resulted in a significantly increased level of thioredoxin-dependent peroxide reductase (PRDX3), a protein with antioxidant function. By comparing the healthy Control group with the DCA group, we observed a significant change only at the level of ATP synthase subunit g (ATP5L) and peroxiredoxin 5 (PRDX5) protein. DCA in control animals induced an upregulation of ATP5L protein and a downregulation of PRDX5 protein. The level of observed proteins in the DM + DCA group approached the conditions of the Control group. We detected ATP synthase F(0) complex subunit B1 (AT5F1), ATP5H, AOFA and PRDX3 as proteins with significantly increased expression and voltage-dependent anion-selective channel protein 3 (VDAC3) with significantly decreased expression in the healthy Control group compared to the DM + DCA group.

#### Protein–protein interactions—within-group comparison

**Figure 6:**
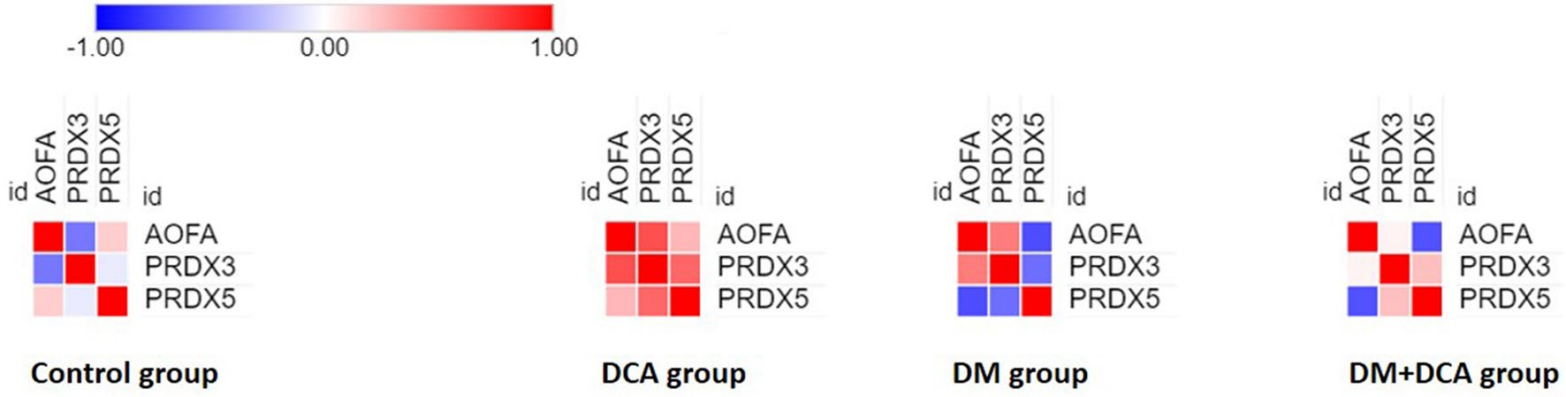
Pairwise Pearson´s correlations between the identified proteins linked to oxidative damage/antioxidant potential visualized as heatmaps stratified by experimental conditions. The color assigned to a point in the heat-map grid indicates the strength of a particular correlation between two proteins. The degree of correlation is indicated by red for positive correlations and blue for negative correlations, as depicted in the color key.

**Figure 7:**
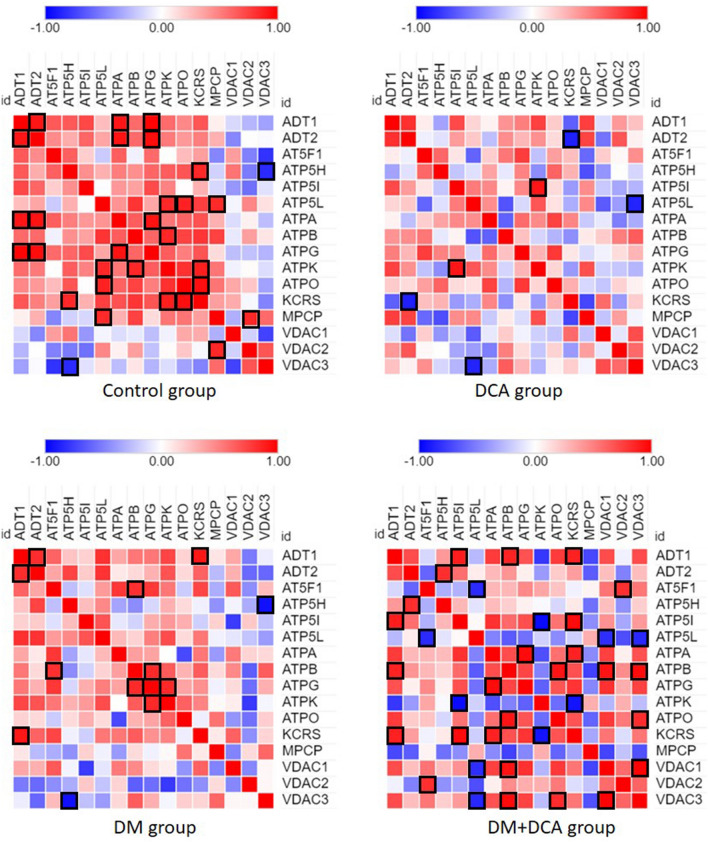
Pearson´s correlation heatmaps between the identified proteins involved in the structure and regulation of mPTPs stratified by experimental conditions. The color assigned to a point in the heat-map grid indicates the strength of a particular correlation between two proteins. The degree of correlation is indicated by red for positive correlations and blue for negative correlations, as depicted in the color key. Statistically significant correlations are marked using cell borders. The upper triangle half of the correlation matrix is axially symmetric along the diagonal to the lower triangle half.

After identifying changes in abundance and expression of the mPTP components and AOFA proteins with peroxiredoxins, we aimed to estimate mutual relationships among the analyzed proteins within the experimental groups. Coexpression analysis helped us to identify protein subgroups that are expressed in a similar fashion. Figures 6 and 7 present the resulting protein correlations in the form of heatmaps, allowing immediate visual detection of significant mutual correlations (indicated by cell borders) and the values of Pearson's correlation coefficient (represented by colors). Pairwise correlations and heatmaps revealed different patterns of protein–protein interactions under the individual experimental conditions.

Figure 6 displays the interactions between AOFA protein associated with ROS production in the heart and PRDX3 and PRDX5 with antioxidant function. Mutual relationships did not show significant correlations. The effect of DCA in the healthy Control group was manifested in these proteins by a change in the direction and strength of the relationship between AOFA-PRDX3 and PRDX3-PRDX5 compared to the Control group. DCA in combination with DM conditions had the opposite effect on these protein interactions, i.e., the positive correlation between AOFA-PRDX3 and the negative correlation between PRDX3-PRDX5 was attenuated. Interactive heatmaps of the mPTP (Fig. 7) proteins indicate positive correlations between ATP synthase subunits, ADT and KCRS in the healthy Control group. However, administration of DCA to the Control group suppressed these interactions, and the number of significant correlations also decreased. Looking at the heatmaps of the Control and DM groups (Fig. 7), we observed a pattern of similarity among the correlation structures for these two conditions, but the strength of the relationship between the proteins was suppressed in the DM group. The interactions of ADT1-ADT2 and ATP5H-VDAC3 proteins remained significant even under pseudohypoxic DM conditions. In the DM + DCA group, the VDAC1 and VDAC3 proteins showed several significant interactions, including a strong positive correlation. VDAC1 formed a significant interaction with ATP5L and ATPB proteins. VDAC3 showed the same interactions as the VDAC1 subunit but also appeared to have a significant positive correlation with the ATPO protein. The DM + DCA group displayed differences in the correlation patterns in comparison to the other groups but also the highest number of significant interactions.

## Discussion

Disorders at the level of oxygen metabolism represent a high risk of developing organ dysfunction. In particular, the myocardium, which is characterized by increased energy requirements under pathological load conditions, is at risk of damage^38^. PC has proven to be an effective cardioprotective tool that ensures energy sustainability by stimulating processes that help increase the tolerance of the myocardium to I/R injury^39–42^. In the present study, we aimed to expand knowledge about the adaptive response of myocardium to the condition of energy load induced by DM. In operational terms, we assume that the condition of experimental DM stimulates myocardium, which could be proven on the level of measurable changes in features linked to regulation of the mPTP opening, ROS signaling and preservation of mitochondrial membrane potential. Our hypothesis was based on the already known facts about the mechanisms of PC that manifested features similar to the state of pseudohypoxia. Application of a short-term load leads to adaptation to more serious pathological stimuli. As one of the possible initiators of endogenous myocardial protection, hypoxia appears to be a potential trigger of adaptation processes^43^. Due to myocardial energy requirements, sites of mitochondrial dysfunction currently appear to be a promising target for cardioprotective therapy^19,44^. The natural and highly unique ability of mitochondrial remodeling in response to pathological stimuli is the key property in the acute stage of the disease leading to the adaptation and compensation of myocardial energy deficiency^45^. For a comprehensive understanding of the mechanisms of signaling pathways and cardioprotective processes regulating energy at the level of isolated cardiac mitochondria, we chose in-depth proteomic analysis in combination with biophysical characterization of the samples. Our findings provide novel insights into the investigated organelles and outline new possibilities for studying their therapeutic potential. The compensatory mechanisms present in DM demonstrated an immediate supply of energy to the myocardium, probably through upregulation of energy production via anaerobic glycolysis^7,46^.

Hormonal and metabolic changes that accompany DM are associated with a decrease in both pyruvate dehydrogenase (PDH) activity and oxidative glucose metabolism. PDHK inactivates PDH, which can lead to adaptive remodeling in the heart accompanied by an increase in fatty acid oxidation capacity and by a decrease in glucose oxidation capacity^46–48^. In connection with the maintenance of cardiac mitochondrial energy through the modulation of pseudohypoxic conditions, the elimination of the preference for anaerobic glycolysis has not yet been explored^49,50^. An effective way to achieve this state is administration of the drug DCA, which acts as a PDHK inhibitor and allows changes in glucose metabolism (Warburg effect)^50,51^. DCA has also the ability to reduce circulating glucose and lactate^52^. The aim of the study was to verify whether the mechanisms of endogenous myocardial protection stimulated by the DM model are enhanced or negated by the effect of DCA or, in other words, whether the DM condition interacts with DCA.

Inhibition of the mPTP opening maintains high oxidative phosphorylation to replenish ATP and creatine phosphate reserves, thus preserving adequate energy production, representing a beneficial cardioprotective strategy^18,22,53^. Detailed proteomic analysis of mPTP, along with selected proteins linked to oxidative damage and those with antioxidant potential, plays an important role in elucidating ways to compensate for cardioprotection. It has provided a significant contribution to the issue of promoting the survival of diabetic myocardium. Previous studies have shown that the pseudohypoxic state of the myocardium is accompanied by increased ROS production^54,55^. The unexpected finding of increased cardiac mitochondrial fluidity in rats with DM, despite the increase in ROS production assumed to be linked to membrane rigidization rather than fluidization, points to the ROS signaling role^56^. In connection with ROS signaling, we supplemented our previous findings at the level of oxidized forms of coenzymes Q_9_ and Q_10_^55^ as well as conjugated dienes^56^ with analysis of AOFA protein levels. The mitochondrial enzyme AOFA is an important source of ROS in the heart, terminating noradrenaline signaling in the heart and forming the H_2_O_2_ byproduct during degradation^57,58^. Increased ROS production results from increased AOFA expression at the transcriptional or posttranscriptional level. Its expression increases naturally during aging but also in pathological conditions such as DM or hypertension. ROS produced by AOFA accumulate in mitochondria mostly in the form of 4-hydroxynonenal (4-HNE). 4-HNE is a product of lipid peroxidation and a reactive aldehyde, which is particularly harmful because it has a longer lifetime than ROS and because it can form adducts with proteins, thereby modifying their function, structure and conformation^59^. AOFA activation and H_2_O_2_ production lead to cardiolipin peroxidation and accumulation of 4-HNE in the mitochondria. 4-HNE specifically binds to voltage-dependent anion-selective channel protein (VDAC) and calcium uniporter protein (MCU), thus regulating Ca^2+^ entry into the mitochondria as a result of AOFA activation^57^. AOFA is also one of the most regulated proteins, as confirmed in various models of rat heart failure induced by volume overload^60^, pressure overload^61^, or myocardial infarction^62,63^. The results of our current experiments on diabetic myocardium confirmed the increased protein level of AOFA compared to the control condition, but after administration of DCA in the DM group, there was a decrease in AOFA expression, which indicates a positive effect of DCA on reducing ROS production. To investigate connection patterns involving regulation of cardioprotective mechanisms and ROS signaling, we included peroxiredoxin proteins in the analysis. These relatively highly abundant proteins with antioxidant functions exhibit both peroxidase and chaperone activity and were found to participate in redox signaling^64^. Mitochondrial peroxiredoxins include PRDX3 and PRDX5^30^. PRDX3 catalyzes the reduction of H_2_O_2_ and organic hydroperoxides, thus providing protection against oxidative damage in the mitochondria in the ischemic myocardium and preventing remodeling of the left ventricular failure due to myocardial infarction^64,65^. Similarly, PRDX5 inhibits endogenous or exogenous peroxide accumulation, thereby acting cytoprotectively^66^. Eecken's study demonstrated the existence of compensatory mechanisms in mitochondrial pig liver samples, where loss of mitochondrial PRDX5 correlated with high expression of PRDX3 and glutathione peroxidase 4^67^. This finding is consistent with results from our analysis, where DCA in the DM group significantly increased expression only at the level of PRDX3 when compared to the DM group. A similar result was observed when we compared the Control group with the DCA group—DCA significantly reduced PRDX5 expression and increased PRDX3 protein levels. Therefore, we can consider DCA to have a stimulatory effect on the PRDX3 protein. The authors Kaludercic and Di Lisa suggested that AOFA activation is associated with the loss of mitochondrial membrane potential, which also corresponds with our achieved results^68^. We observed a decrease in the membrane potential in the DM group, presumably due to the increase in ROS. In addition to the stimulation of PRDX3 protein possessing antioxidant activity and to the reduction in AOFA levels, the effect of DCA and diabetic conditions (DM + DCA) probably caused the decrease in ROS production. In turn, it promoted the increase in mitochondrial membrane potential relative to the DM group, thus approaching the values obtained for the healthy Control group.

Interestingly, DCA administered to control animals, on the other hand, induced depolarization and a decrease in the mitochondrial membrane potential. This finding has also been reported by other authors^69^. These observations suggest that the positive effect of DCA occurs only under conditions of increased energy load, which is a hallmark of the DM model. Our previous results demonstrating a significant increase in mitochondrial membrane fluidity in the DM myocardium might also be related to mPTP regulation^70^. mPTP opening may result in the depolarization of membranes and subsequent disconnection of oxidation from oxidative phosphorylation. This, however, has not been confirmed in our previous experiments^6,71^.

Despite the reduced membrane potential and the increased level of AOFA protein, which is considered to be a known source of ROS in the heart, no decrease in mitochondrial CRC was detected in the DM group, which also corresponds to maintaining or increasing the expression of the analyzed proteins forming and regulating mPTP. Diabetic condition is also associated with increased fatty acids oxidation that provokes alterations of mitochondrial functions in the heart and impairs mitochondrial bioenergetics and Ca^2+^ homeostasis^72^. It is also discussed that mitochondria from Type 1 diabetic heart have depressed capacity to accumulate Ca^2+^, because of an enhanced sensitivity to induction of mPTP opening^73,74^. In our study the generated amount of ROS and increased fatty acid oxidation were probably not as harmful to the mitochondria to cause mPTP opening to become out of control. Under our experimental conditions, we observed a significant decrease in CRC in the DM group treated with DCA in comparison to the other experimental groups. However, in the DM + DCA group, the AOFA protein was downregulated compared to that in the DM group, and the transmembrane potential was maintained at the control level. Although DCA in combination with DM conditions was associated with a decrease in radicals, the joint effect of DCA and DM probably caused elimination of the positive signaling by which the radicals initiated endogenous protection and subsequent adaptation to a pathological stimulus induced by experimental DM. Among all experimental groups, the DM group exhibited stimulation to the highest aggregated abundance of the mPTP proteins, as revealed in proteomic analysis. Administration of DCA to the DM group reduced the aggregated abundance of mPTP proteins almost to the level of the healthy Control group. The comparison of the DM to Control groups showed significantly increased levels of 3 ATP synthase subunits and VDAC1 protein in a total of 17 quantified mPTP-forming and regulating proteins. VDAC has been shown to regulate Ca^2+^ uptake through the outer mitochondrial membrane. Furthermore, VDAC1 overexpression supported Ca^2+^ intake by mitochondria^75^. Sasaki et al. reported that elevated VDAC1 levels in diabetes lead to mitochondrial Ca^2+^ overload and subsequently to an increased rate of apoptosis^76^. Downregulation of the VDAC1 isoform is a part of the cardioprotective pathway during I/R injury. Elevated VDAC1 levels in the studied D model are probably due to increased Ca^2+^ entry into mitochondria, which may also be related to a decrease in the mitochondrial membrane potential and to an increase in ROS production. Increased Ca^2+^ entry is one of the pathological mechanisms that play an important role in the signaling leading to the onset of endogenous myocardial protection effective against increased hypoxic burden^77–80^. Administration of DCA to diabetic animals caused a significant decrease in VDAC1 and VDAC3 protein expression. This result achieved by proteomic analysis suggests a positive effect of DCA on Ca^2+^ regulation.

ATPA is one of the most abundant proteins among the quantified mPTP proteins^53^. It can be assumed that even changes in this single ATP synthase subunit might represent a sufficiently strong regulator of energy production through the ATP synthase complex. ATPA expression was significantly increased in the DM group, and in the DM + DCA group, it decreased to the Control level. This finding supports our previous results from studies using an experimental model of diabetic myocardium, where the myocardium is able to maintain increased ATP synthase activity by stimulating endogenous protective mechanisms^6^. Also, we demonstrated that total adenine nucleotide contents ATP and ADP were non-significantly decreased in diabetic hearts. These findings indicated a slight but constant degree of energy deficiency in acutely diabetic myocardium that can in a great part originate in depletion of the total adenine nucleotides content^6^.

By performing a deep inspection of the interactive heatmaps, we confirmed the observation from the previous study by our group, which showed that despite only subtle changes in the protein levels, different patterns of mutual protein interactions can be identified across the studied experimental groups^53^. We found that the effect of DCA in the Control group manifested positive correlations between all 3 identified mitochondrial proteins associated with ROS production or, conversely, with antioxidant function. DCA in the DM group significantly reduced AOFA levels and increased PRDX3 levels. However, the interaction between these proteins based on the correlation heatmap outputs has not been demonstrated. Protein–protein interactions of the mPTP complex in the Control group revealed a positive correlation between ADT subunits together with ATP synthase subunits and KCRS; however, administration of DCA disrupted these interactions. Comparison of the heatmaps of the DM and Control groups showed a weakening of the protein interactions under pseudohypoxic conditions. The expression levels of VDAC1 and VDAC3 proteins were significantly reduced after DCA administration in the DM group and manifested in several significant interactions in the heatmaps of the DM + DCA group, including the mutually significant interaction between these proteins.

The experimental DM model elicited a positive response based on proteomic data as well as on membrane modulations of cardiac mitochondria and regulation of mPTP opening, resulting from stimulation of endogenous myocardial protection and effective adaptation. VDAC1 upregulation, as a response to increased Ca^2+^ entry into mitochondria, might indicate Ca^2+^ signaling. This finding, together with the ROS signaling function, can be considered one of the important initiators of adaptive cardioprotective mechanisms. The obtained results are supplemented by original findings of protein interactions, which indicate the presence of significant positive correlations between ATP synthase subunits in healthy animals. Additionally, an identical pattern of interactions was observed in the diabetic myocardium, but the interactions were attenuated. Treatment with DCA in combination with DM regulated ROS-related proteins, increased the mitochondrial membrane potential and eliminated increased Ca^2+^ entry in comparison with the DM condition. In terms of protein interactions between mPTP proteins, the pattern of ATP synthase subunit interactions was disrupted, but DCA clearly acts as a significant effector of VDAC protein interactions. Therefore, it can be concluded that DCA, despite the elimination of the DM-induced endogenous protective mechanism, provided a sufficiently strong positive response and protected the energy-loaded myocardium.

In summary, DCA has been shown to be an effective modulator of cardiac mitochondrial signaling pathways, acting via its own mechanism and providing benefits in the myocardium of diabetic rats. The effect of DCA in combination with DM appears to be particularly supportive in the regulation of ROS-related proteins, which is associated with the maintenance of membrane potential, but without increasing the resistance of mPTP to Ca^2+^ overload. Both DM and DCA exhibit an individual cardioprotective pathway. Their combination does not provide an additive effect. The achieved results significantly contributed to the understanding of the mechanisms leading to the maintenance of myocardial energy balance and expanded the available knowledge about mitochondrial signaling pathways in the rat heart and their modulations that can lead to cardioprotection.

## Materials and methods

### Experimental animals

All animal experiments were conducted in accordance with the rules issued by the State Veterinary Administration of the Slovak Republic, legislation No 377/2012 and with the regulations of the Animal Research and Care Committee of Centre of Experimental Medicine SAS—Project No. 2237/18–221/3, approved on 21 August 2018. This study was carried out in accordance with recommendations in the ARRIVE guidelines.

Male Wistar rats (Dobrá voda, Slovakia) aged 12–15 weeks were used for our experiments. Animals were randomly divided into 4 experimental groups: (i) healthy control group (Control), (ii) group affected by acute streptozotocin-induced diabetes mellitus (DM), (iii) healthy control group with administration of metabolic regulator dichloroacetate (DCA), and (iiii) group with acute streptozotocin-induced diabetes mellitus modulated with DCA (DM + DCA). Experimental animals were housed under natural mode conditions (22 ± 2 °C, humidity 55 ± 10%, 12:12 dark/light cycle) with constant access to drinking water and standard pellet diet ad libitum*.*

### Experimental models

An experimental model of acute diabetes was induced in male Wistar rats using a single intraperitoneal injection of streptozotocin (65 mg/kg of body weight, Sigma–Aldrich, USA) dissolved in 0.1 M citrate buffer (pH 4.0) 8 days prior to the experiment. Beginning from induction until the onset of the acute phase, glycosuria was monitored daily using GlukoPHAN strips (Pliva–Lachema, Brno, Czech Republic). Blood glucose, cholesterol and triacylglycerols were measured (MultiCare and appropriate test strips, Biochemical System International, Florence, Italy) on the eighth day after streptozotocin administration using blood collected from the tail vein as well as insulin in the serum (RIA kit, Linco Research, USA). Animals that revealed glycemia ≥ 20 mmol/L were considered diabetic.

DCA (Sigma–Aldrich, USA) was administered to animals intraperitoneally in two doses (150 mg/kg and 75 mg/kg) 60 min and 15 min before heart excision^81^. DCA acts as an inhibitor of PDHK and ensures the activation of PDH, which is normally inhibited in the diabetic myocardium.

### Administration of anesthesia

The experiment was performed on intraperitoneally anesthetized animals (thiopental, 50–60 mg/kg of body weight applied with heparin 500 IU). Prior to heart extraction, the animals were stabilized for 30 min.

### Isolation of cardiac mitochondria

Mitochondria were isolated from rat hearts; immediately after extraction from the chest, hearts were immersed in cooled saline solution (0.9% NaCl, 4 °C) and washed to remove blood residues, aorta and fat. Differential centrifugation was used for mitochondrial isolation at 4 °C. After adding a small amount of cooled isolation solution (180 mM KCl, 4 mM ethylenediaminetetraacetic acid (EDTA) and 1% bovine serum albumin dissolved in dH_2_O, pH 7.4), the heart was milled with the GentleMACS Octo Dissociator (Miltenyi Biotec GmbH, Germany) (2 cycles per 1 min). Subsequently, cooled isolation solution was added to the sample up to 20 mL. This solution was homogenized using a manual homogenizer and centrifuged (1000 g, 10 min). The obtained mitochondria-containing supernatant was repeatedly centrifuged (6200 g, 10 min). The resulting sediment was suspended in a homogeneous state in 20 mL of cold isolation solution without albumin (180 mM KCl and 4 mM EDTA dissolved in dH_2_O, pH 7.4) and centrifuged again (6200 g, 10 min). The sediment (resulting mitochondrial fraction) was diluted with a small amount of isolation solution without albumin and homogenized^11^. The concentration of proteins in the mitochondrial fraction was determined by a Synergy H1 multidetection reader (BioTek, USA) using Bradford’s method^82^.

**Figure 8:**
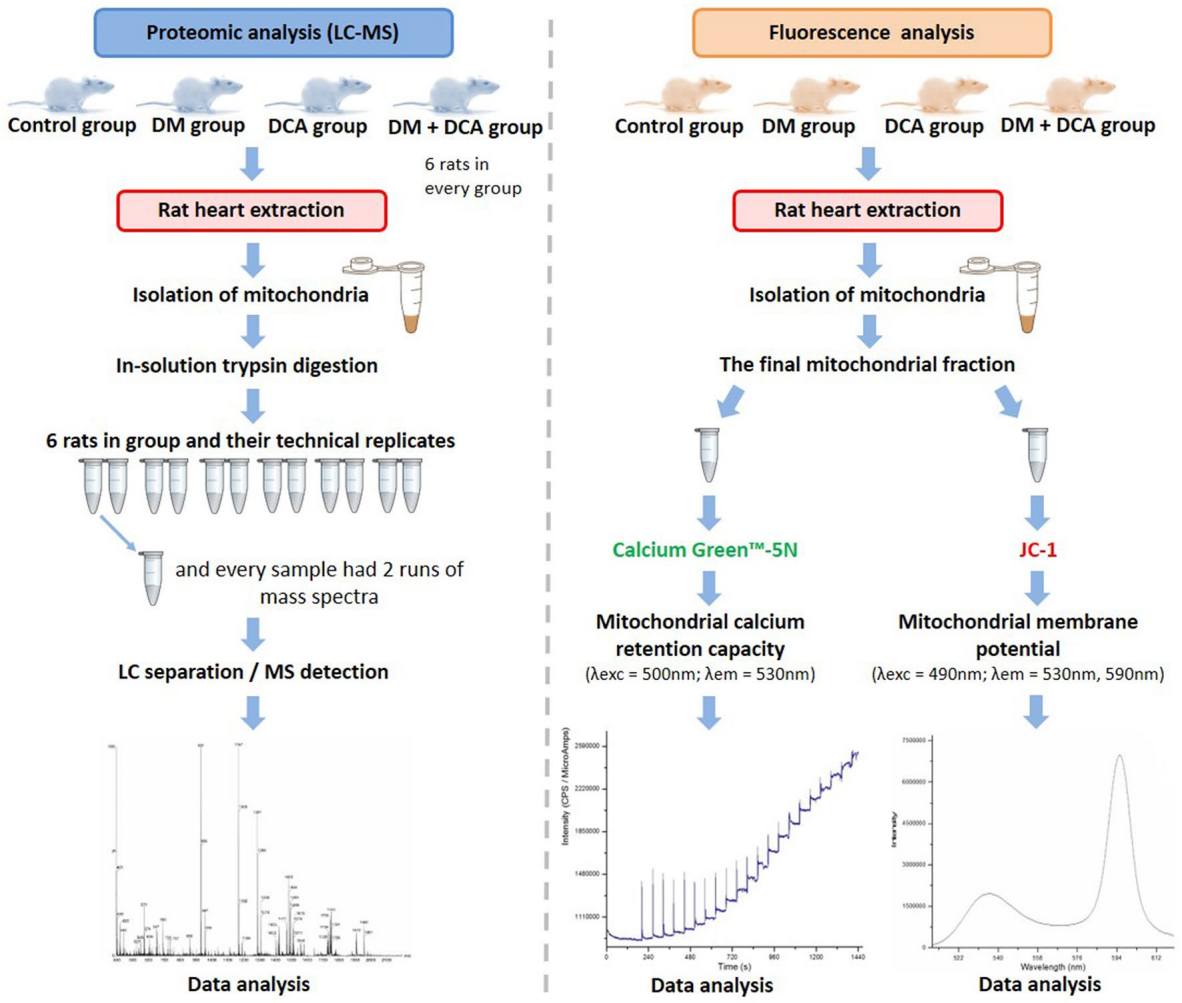
Overview of the experimental workflow. This figure was made in Microsoft PowerPoint (Microsoft PowerPoint for Microsoft 2016 MSO (Version 2207 Build 16.0.15427.20166), Microsoft.com).

A schematic overview of the methodological procedures can be seen in Fig. 8.

### Measurement of mitochondrial membrane potential using the fluorescent dye JC-1

The freshly isolated mitochondrial suspension was diluted to a concentration of 1 mg protein/ml with cooled 20 mM MOPS buffer (3-(N-morpholino) propane sulfonic acid), pH 7.5, containing 110 mM KCl, 10 mM ATP, 10 mM MgCl_2_, 10 mM sodium succinate, and 1 mM EGTA. Samples were kept on ice until measurement. The mitochondrial membrane potential was analyzed using a FluoroLog FL3 spectrofluorometer (HORIBA Scientific, USA) and the fluorescent dye JC-1 (5,5′,6,6′-tetrachloro-1,1′,3,3′-tetraethylbenzimidazolylcarbocyanine iodide, Enzo Life Sciences). The measurement was performed in the cuvette using a diluted suspension of 0.25 mg mitochondrial protein/ml 20 mM MOPS buffer with pH 7.5, while the resulting volume was 2 ml. JC-1 (2 µl, 0.3 mM) was added to the suspension, and the fluorescence intensity of the membrane potential was measured after 10 min of incubation at 25 °C. The fluorescence of each mitochondrial sample was monitored using an excitation wavelength of 490 nm and two emission wavelengths of 530 nm and 590 nm simultaneously. JC-1 represents a lipophilic cationic dye that is able to enter and redistribute in mitochondria depending on membrane potential; it can be in the form of monomers or J-aggregates. While JC-1 dominates in depolarized mitochondria in the form of monomers that emit green fluorescence (~ 530 nm), in energized and negatively charged mitochondria, it dominates in the form of J-aggregates that emit red fluorescence (~ 590 nm)^83^. Next, 10 µl of 2,4-dinitrophenol (54.32 mM) was added, and the fluorescence was measured after 2 min of incubation at 25 °C. 2,4-dinitrophenol increases the proton permeability of the inner mitochondrial membrane, leading to the depolarization of membrane potential, which served as a positive control^84^. The data are presented as a ratio of red J-aggregates and green monomers.

### Measurement of the mitochondrial calcium retention capacity

For the monitoring of changes in extramitochondrial Ca^2+^ concentration and the ability of mitochondria to buffer exogenous Ca^2+^, a CRC assay modified by Gomez et al., Duicu et al. and Harisseh et al. was used^85–87^. Fresh myocardial mitochondria were suspended in 2 ml of cooled incubation CRC buffer (50 µg protein/ml incubation CRC buffer composed of 150 mM sucrose, 50 mM KCl, 2 mM KH_2_PO_4_, 5 mM succinic acid in 20 mM Tris–HCl, pH 7.4 in 37 °C). The fluorescent probe Calcium Green™-5 N (10 µl of 0.1 mM, Invitrogen, excitation-emission, 500–530 nm) was added to the diluted mitochondrial suspension, while the resulting volume in the cuvette was 2 ml. After consequent stabilization (200 s), 0.125 mM CaCl_2_ was added at regular intervals (60 s) in a constant amount of 4 µl at room temperature with stirring. The change in the extramitochondrial concentration of Ca^2+^ was continuously monitored using a FluoroLog FL3 spectrofluorometer (HORIBA Scientific, USA). The fluorescent probe Calcium Green™-5 N has the characteristics of a low affinity impermeable dye that shows increased intensity of fluorescent emission after binding to Ca^2+^, while the probe does not fluoresce if Ca^2+^ is not present^88^. Calcium Green™-5 N also has a higher dissociation constant; thus, it serves as an appropriate indicator for monitoring the kinetics of the fast dynamics of Ca^2+^^89^. As a result of CaCl_2_ addition, an increased fluorescent signal associated with an increased Ca^2+^ concentration in the extramitochondrial space was detected. The signal decreased after uptake of Ca^2+^ to the mitochondrial matrix to almost baseline values. This effect was observable until mPTP opening due to Ca^2+^ overload in the mitochondrial matrix, shown as a sudden rise in extramitochondrial Ca^2+^ concentration accompanied by an increased fluorescent signal. Therefore, the amount of CaCl_2_ needed for the induction of mPTP opening serves as an indicator of the sensitivity of mPTP to Ca^2+^ overload in experimental models simulating pathological burden.

### Proteomic analysis by nanoliquid chromatography and mass spectrometry (LC–MS/MS)

Mitochondrial samples containing 250 μg of protein were digested by trypsin in solution (Sigma–Aldrich, USA) at a ratio of 1:25 (a concentration of trypsin 0.2 µg/µL) overnight at 37 °C, and sample preparation was identical to that reported previously^53^. Before proteomic analysis using a Nano System liquid chromatograph (Ultimate 3000 RSLC, Thermo Fisher Scientific, Germering, Germany) followed by mass spectrometry with electrospray ionization (ESI) and a 3D ion trap mass analyzer (Amazon SL, Bruker, Bremen, Germany), samples were desalted by using C18-U SPE columns (Strata, Phenomenex). All LC–MS parameters and database searching and protein identification methods were described previously^53^.

### Statistical analyses and interpretation

Descriptive and univariate analyses were performed on all selected animals' characteristics. Mean ± SEM (standard error of mean) is given for the normally distributed variables or a median and interquartile range if data showed substantial deviations from normality. Differences between the groups were tested with one-way analysis of variance (ANOVA), followed by the Dunnett test for multiple comparisons with the control or Tukey–Kramer test for all pairwise comparisons. In the case of nonnormality and/or unequal variances between groups being compared, a nonparametric alternative (the Kruskal–Wallis test and the post hoc pairwise comparisons with the Connover-Inman test) was performed. We used a two-way ANOVA to analyze data from factorial experiments: DCA treatment as the first main factor and experimentally induced diabetic condition as the second main factor.

Analysis of proteomic data was performed according to the model presented previously in Andelova et al.^53^. In our analysis, we focused on mitochondrial proteins, which are considered structural and regulatory components of the mPTP complex, and 3 proteins related to oxidative damage or antioxidant potential, which occurred at the intersection of all 4 groups. Proteins with two or more identified peptides were further evaluated by the label-free method of the exponentially modified Protein Abundance Index (emPAI). The retrieved emPAI values were used to create a so-called “fold change” (FC) measure. The FC was defined as the ratio of abundances under 2 different experimental conditions averaged across replicates under these conditions. The log-transformed value of the 2 experimental group ratios—individually for each protein—was tested against the null hypothesis of no change. The absolute FC was calculated as 2^log^_2_^value^. Two-way repeated-measures ANOVA was used to verify the treatment effect. To evaluate differences in abundance levels of quantifiable proteins between two different experimental conditions, a two-sample t test was performed on log-transformed technical replicates. To estimate mutual relationships among the identified proteins within the groups, coexpression analysis was performed. Exploratory data analysis and using Pearson's correlation coefficient served for visual inspection of mutual correlation with significance. The resulting correlations presented in the form of heatmaps were obtained with Morpheus online software (https://software.broadinstitute.org/Morpheus/).

All *p value*s were considered statistically significant at a two-tailed *p value* of < 0.05. For more detailed information, see Andelova et al.^53^.

Statistical analyses were performed using StatsDirect 3.0.191 software (Stats Direct Ltd., Cheshire, UK), Statistica 13 software (Dell-StatSoft, Inc. Tulsa, OK, TIBCO Software Inc. USA) and GraphPad Prism 8.0.1 (GraphPad Software, Inc., USA).

## Data Availability

The mass spectrometry proteomics data have been deposited to the ProteomeXchange Consortium via the PRIDE^90^ partner repository with the dataset identifier PXD033256 on https://www.ebi.ac.uk/pride/archive/projects/PXD033256.
